# Rapid Processing of Both Reward Probability and Reward Uncertainty in the Human Anterior Cingulate Cortex

**DOI:** 10.1371/journal.pone.0029633

**Published:** 2011-12-27

**Authors:** Rongjun Yu, Wu Zhou, Xiaolin Zhou

**Affiliations:** 1 Department of Psychology, South China Normal University, Guangzhou, China; 2 Department of Psychology and Center for Brain and Cognitive Sciences, Peking University, Beijing, China; 3 Departments of Otolaryngology and Communicative Sciences, Neurology and Anatomy, University of Mississippi Medical Center, Jackson, Mississippi, United States of America; 4 Key Laboratory of Machine Perception, Ministry of Education, Peking University, Beijing, China; University of British Columbia, Canada

## Abstract

Reward probability and uncertainty are two fundamental parameters of decision making. Whereas reward probability indicates the prospect of winning, reward uncertainty, measured as the variance of probability, indicates the degree of risk. Several lines of evidence have suggested that the anterior cingulate cortex (ACC) plays an important role in reward processing. What is lacking is a quantitative analysis of the encoding of reward probability and uncertainty in the human ACC. In this study, we addressed this issue by analyzing the feedback-related negativity (FRN), an event-related potential (ERP) component that reflects the ACC activity, in a simple gambling task in which reward probability and uncertainty were parametrically manipulated through predicting cues. Results showed that at the outcome evaluation phase, while both win and loss-related FRN amplitudes increased as the probability of win or loss decreased, only the win-related FRN was modulated by reward uncertainty. This study demonstrates the rapid encoding of reward probability and uncertainty in the human ACC and offers new insights into the functions of the ACC.

## Introduction

Reward probability and uncertainty are essential parameters in the computation of the utility function of a behavior choice [1], [2]. Whereas reward probability crucially determines the expected reward value associated with a behavior choice, reward uncertainty, i.e., the variance of the probability distribution, provides an estimate of the risk associated with the same choice. In non-human primates, substantial evidence indicates that the midbrain dopamine neurons encode the reward prediction signal that is based on reward probability, as well as the reward prediction error signal that is the difference between the actual and expected reward [3]–[5]. The cues that predict higher reward probabilities evoke larger phasic activations in the midbrain dopamine neurons. Whereas the outcomes that are better than predicted (positive prediction errors) evoke phasic activations in the dopamine neurons, the outcomes that are worse than predicted (negative prediction errors) evoke phasic inhibitions. In a seminal study, Fiorillo et al. (2003) further showed that the midbrain dopamine neurons encode reward uncertainty in their tonic discharges. Recent fMRI studies reported similar encoding of reward probability and uncertainty in the human midbrain regions [6], [7].

The anterior cingulate cortex (ACC) receives projections from the midbrain dopaminergic regions and has been proposed to play an important role in reward processing. Event-related potential (ERP) studies in humans found that an ERP component, called the feedback related negativity (FRN), is sensitive to reward expectation error. The FRN, which peaks at around 300 ms and is maximal at frontal-central scalp electrode sites, is likely being generated in the ACC [8]–[10]. Consistent with this account, fMRI studies of ACC have shown that the activity in ACC can reflect reward prediction errors [11], [12]. A recent fMRI study also found that the ACC activity is modulated by the uncertainty of reward environment during feedback monitoring and the degree of such modulation predicts the learning rate across individuals [13], suggesting that the ACC may track the reward uncertainty.

The goal of this ERP study is to use the FRN amplitude as a measure of the ACC activity and perform a quantitative analysis of the encoding of both reward probability and uncertainty in the ACC. As the uncertainty is derived and calculated from the probability [14], in most circumstance, these two factors are highly correlated. Increasing the probability of win from 75% to 100% not only changes reward probability but also decreases uncertainty (i.e., 100% win is most certain). On the other hand, decreasing the probability of win from 25% to 0% not only decreases reward probability but also decreases the uncertainty (i.e., 0% win is most certain). The uncertainty reaches its maximum when reward probability is 50%. Above 50%, it decreases as reward probability increases, whereas below 50%, it decreases as reward probability decreases. Given these opposite directions of correlations, the correlation between probability and uncertainty will be close to zero if the win probability varies from 0 to 100%. In this study, to ensure reward probability and uncertainty could be disassociated, reward probability was varied over a wide range of probabilities with a sufficient number of intermediate values (every 12.5% from 0 to 100%). Given the evidence that the ACC encodes both reward probability and uncertainty in fMRI and the evidence for the link between the FRN and the ACC [8]–[13], we predicted that the FRN amplitude would be modulated by both reward probability and uncertainty.

## Materials and Methods

### Participants

Sixteen undergraduate students (8 male; mean age 22±2.5 years) participated in the gambling experiment. They were told that their performance in the gambling task determined how much they would be awarded or penalized on the top of a base payment of 40 yuan (about US $6). Written, informed consent was obtained from each participant, and the study was approved by the Academic Committee of the Department of Psychology at Peking University.

### Experimental design

**Figure 1:**
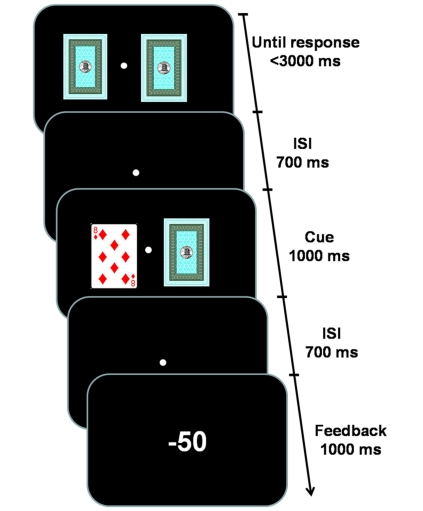
Illustration of events and timing in a single trial.

We used a modified version of a gambling task in which reward probability and uncertainty were manipulated parametrically [14]–[16] (Fig. 1). In each trial, participants were first presented with the back side of two cards that were drawn without replacement randomly from a deck of nine cards numbered between 2 to 10. They were asked to guess within 3000 ms which card had a larger number in order to win 0.5 yuan. A 0.5 yuan penalty was imposed for late response. Participants were explicitly informed about this rule and a visual feedback “too late, lose 0.5 yuan” was presented to participants if they failed to respond within 3000 ms. At 700 ms after participants' response, the chosen card (called cue card) was presented for 1000 ms. The winning probability was indicated by the number of the cue card ranging from 2 to 10, which corresponded to the winning probability of 0, 0.125, 0.25, 0.375, 0.5, 0.675, 0.75, 0.875, and 1, respectively. Participants were explicitly informed of these probabilities. At 700 ms after the offset of the cue card, a sign of “+50” or “−50” was presented for 1000 ms to indicate a win (and 0.5 yuan reward) or loss (and 0.5 yuan penalty) trial, respectively. We only presented the numeric feedback without showing the original two cards in order to control for the visual property of feedback stimuli. The next trial began 1000 ms after the offset of the feedback in the previous trial. The experiment consisted of 9 blocks of 96 trials with each cue card being presented a total of 96 times. For each cue condition, the proportion of trials for the win or loss outcome followed exactly the probability indicated by the cue number. For example, for the cue card 3, 12.5% trials would give the win feedback and 87.5% trials the loss feedback. There was a short break between blocks.

For each condition, reward probability was indicated by the number in the cue card, as we pointed out earlier. There are several measures of uncertainty that are all maximal at P = 0.5 and minimal at P = 0 or 1. In this study, uncertainty was defined as reward variance, which is an inversely quadratic function of probability [14], [16]. Thus, reward uncertainty has a value of 0, 0.44, 0.75, 0.94, 1, 0.94, 0.75, 0.44 and 0 for reward probability value of 0, 0.125, 0.25, 0.375, 0.5, 0.625, 0.75, 0.875 and 1, respectively (Table 1). We used this measure in order to be consistent with previous neuroimaging studies [14], [16]. Uncertainty was also measured as entropy [4] and similar effects were observed. At the outcome stage, positive prediction error elicited by actual win feedback was measured as 1 minus probability of winning at the cue stage, whereas negative prediction error elicited by actual loss feedback was measured as 1 minus probability of losing at the cue stage (see Table 1). The uncertainty prediction error was measured as the uncertainty at the cue stage minus 0 as there was no uncertainty at the outcome stage (uncertainty resolved). At the cue stage, two analyses were carried out: a one-factor ANOVA analysis with 9 levels of probability and a regression analysis with mean FRN amplitudes across participants as dependent variable and reward probability and reward uncertainty as two independent variables. Repeated measures ANOVA analyses tested whether the FRN amplitude showed significant linear or quadratic relationship with reward probability. Since uncertainty, measured as reward variance, is an inversely quadratic function of probability that is minimal at P = 0 and P = 1 and maximal at P = 0.5, a significant quadratic effect would suggest a significant relationship between uncertainty and FRN amplitude. Degrees of freedom were corrected using Greenhouse-Geisser estimates of sphericity when the Mauchly's test indicated that the assumption of sphericity had been violated. Note, because uncertainty was calculated from probability, it was impossible in ANOVA to examine both factor together, for example, controlling probability while examining the effect of uncertainty. While the ANOVA analyses examine the effect of probability or uncertainty separately, linear regression analyses examine the effect of one factor (e.g. probability) after controlling for the all other factors in the model. Similar data analyses were carried out for the FRN at the outcome stage.

**Table 1:** The win probability and uncertainty for each of the nine conditions at the cue phrase and the reward prediction error and uncertainty prediction error associated with win and loss outcomes.

Cue phase				Actual wins in the outcome phase			Actual losses in the outcome phase		
Cue number	Win probability	Uncertainty	FRN amplitude	Positive PE	UncertaintyPE	FRN amplitude	Negative PE	Uncertainty PE	FRN amplitude
2	0	0	−1.859	N/A	N/A	N/A	0	0	−1.252
3	0.125	0.438	−1.785	0.875	0.438	−2.696	0.125	0.438	−0.854
4	0.25	0.75	−2.346	0.75	0.75	−1.907	0.25	0.75	−0.743
5	0.375	0.938	−1.749	0.625	0.938	−0.807	0.375	0.938	−1.487
6	0.5	1	−1.985	0.5	1	−0.532	0.5	1	−2.617
7	0.625	0.938	−1.876	0.375	0.938	−0.044	0.625	0.938	−3.27
8	0.75	0.75	−1.641	0.25	0.75	−0.743	0.75	0.75	−3.943
9	0.875	0.438	−1.667	0.125	0.438	−0.838	0.875	0.438	−4.232
10	1	0	−0.889	0	0	−1.338	N/A	N/A	N/A

Grand mean FRN amplitudes (µV) during the interval 275–325 ms post-cue across participants are also presented. PE = prediction error.

### ERP recording and analysis

EEGs were recorded from 64 scalp sites using tin electrodes mounted in an elastic cap according to the International 10/20 system (NeuroScan Inc. Herndon, Virginia, USA). The impedance of electrodes was maintained below 5 KΩ. Eye blinks were recorded from the left supraorbital and infraorbital electrodes. The horizontal electro-oculogram (EOG) was recorded from electrodes placed 1.5 cm lateral to the left and right external canthi. All electrode recordings were referenced to an electrode placed on the left mastoid. The EEG and EOG were band-pass filtered (0.05∼70 Hz), sampled at 500 Hz and stored in hard disks for off-line analysis.

Ocular artifacts were corrected with an eye-movement correction algorithm (Gratton et al., 1983). All trials in which EEG voltages exceeded a threshold of +/− 70 µV during the recording epoch were excluded from analysis. The EEG data were re-referenced offline to linked-mastoid electrodes by subtracting 50% of the signal in the right mastoid electrode from the signal in each channel. The EEG signal was baseline corrected and further band-pass filtered from 2∼20 Hz (24 dB octave roll off). This was to minimize the overlap between the FRN and other reward-sensitive ERP components, particularly the P300, since it has been known that the P300 is a closely associated slow wave ERP response [17], [18]. Epochs of 800 ms (with 200 ms pre-stimulus baseline) EEG from each electrode were sorted by experimental conditions.

At both cue and feedback phases, the FRN was measured as the mean amplitude at Fz, where there was maximal effect of valence (loss minus win), during the interval 275–325 ms after stimulus presentation [19], [20]. To confirm that our findings were not affected by the particular time window we selected for the FRN, we also reported the mean FRN amplitude during the interval 250–325 ms post-stimulus for cue conditions. The FRN was also measured as the base to peak amplitude and a similar pattern of effects was observed. We did not use the difference wave approach since our aim was to quantitatively evaluate the relationship between the FRN amplitudes and probability or uncertainty rather than simply compare the FRN amplitudes in two experimental conditions [21], [22]. To assess the coding of reward probability and uncertainty by the FRN, linear regressions were performed using the mean FRN amplitude (in each condition) as a dependent variable and reward probability and uncertainty as independent variables.

### Dipole Analysis

An attempt was made to localize the dipole sources of the ERP components at the cue phase and the feedback phase. The cue ERP waveform was generated by averaging all cue locked ERP waveforms across all conditions. The win or loss ERP waveform was generated by averaging all feedback locked ERP waveforms across all win or loss conditions. Source localization was carried out with the Brain Electrical Source Analysis program (BESA, Version, 5.0) using a four-shell ellipsoidal head model. As suggested [23], data were high-pass filtered (2 Hz) before dipole fitting in order to remove slow drifts which could bias the resulting solution.

For both cue and feedback locked ERP components, time windows of 75 to 125, 150 to 200, and 250 to 350 ms post-response, were chosen for the localization analysis of the N1, P2, and FRN components, respectively. We use symmetric dipoles for the localization analysis of the N1 and P2 components since early sensory processes were likely to occur at both hemispheres. The dipoles were fitted with no restriction on their direction and location for each component and then fitted with fixed location for the 0 to 350 interval covering all the ERP components.

## Results

### Cue-evoked FRN

**Figure 2:**
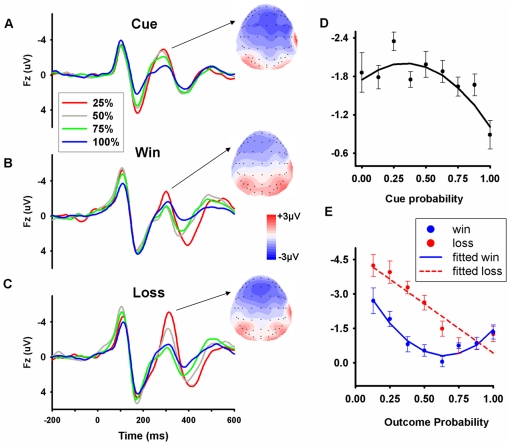
Grand-average ERP waveforms from channel *Fz*. ERPS were time locked to (A) the cue phase, (B) win outcome condition, and (C) loss outcome condition. Please note, the outcome probability used in this figure refers to the actual outcome frequency. Thus low probability indicates that the outcome is infrequent. For example, 25% probability in win condition refers to ‘actual win after the prediction of 25% winning probability’, whereas 25% probability in loss condition refers to ‘actual loss after the prediction of 75% winning probability’. For clarity, only waveforms for probabilities of 25%, 50%, 75%, and 100% are presented. The topographic map of mean FRN at 300ms in the cue, win, and loss conditions were also shown. (D) Coding of reward probability and reward uncertainty in cue-evoked FRN, and (E) outcome-evoked FRN. The regression lines were computed based on the regression equations for each condition.

For the cue-evoked FRN in the interval of 275–325 ms post-cue presentation (Table 1 and Fig. 2A), ANOVA with 9 levels of probabilities revealed a significant main effect of probability, F(8,120) = 3.57, *p* = 0.016, a significant linear main effect, F(1,15) = 5.33, *p* = 0.036, and a marginally significant quadratic effect, F(1,15) = 3.28, P = 0.09. For the cue-evoked FRN in the interval 275–325 ms after cue presentation, similar ANOVA revealed a significant main effect of probability, F(8,120) = 5.874, *p* = 0.001, a marginally significant linear main effect, F(1,15) = 3.977, *p* = 0.065, and a significant quadratic effect, F(1,15) = 10.657, *p* = 0.005. These results suggest that the FRN encodes reward probability, such that smaller reward probability was associated with larger FRN amplitude, as well as reward uncertainty, although these effects are not robust.

Regression analysis on mean FRN amplitudes revealed that the regression coefficient (Beta value) associated with reward probability was 0.745±0.24 and the coefficient associated with reward uncertainty was −0.556±0.21. T tests revealed that both coefficients were significantly different from zero (*t* = 3.08, *p* = 0.022 for probability, and *t* = −2.64, *p* = 0.038 for uncertainty), suggesting that cue-evoked FRN was modulated by both reward probability and uncertainty. The coefficients indicated that the FRN had larger amplitudes for smaller reward probabilities and high uncertainties (Fig. 2D). The proportion of the variance explained by the model was high, with *R* ^2^ = 0.73, *p* = 0.019. Note, the uncertainty effect might be interpreted with caution, as the effect may predominately driven by the P = 1 condition. After taking out the P = 1 condition, there was no significant correlation between FRN amplitude and reward probability or uncertainty (P values >0.05).

For the interval 250–325 ms post-cue (Table 2), regression analysis revealed that both probability coefficient (0.565±0.26) and uncertainty coefficient (−0.789±0.23) were significantly different from zero (t = 2.17, p = 0.073 for probability, and t = −3.33, p = 0.014 for uncertainty). The explanation power was the same as the model on FRN data in the interval of 275–325 ms post-cue.

**Table 2:** The win probability and uncertainty for each of the nine conditions at the cue phrase.

Cuenumber	Winningprobability	Uncertainty	FRNamplitude
2	0	0	−1.859
3	0.125	0.438	−1.785
4	0.25	0.75	−2.346
5	0.375	0.938	−1.749
6	0.5	1	−1.985
7	0.625	0.938	−1.876
8	0.75	0.75	−1.641
9	0.875	0.438	−1.667
10	1	0	−0.889

Grand mean FRN amplitudes (µV) during the interval 250–325 ms post-cue across participants are also presented.

### Outcome-evoked FRN

ANOVA with two types of outcomes (win/loss) and 8 levels of probabilities revealed a significant main effect of valence, F(1,15) = 16.39, P = 0.001, a significant main effect of probability, F(7,105) = 12.91, P<0.001, and a significant interaction between valence and probability, F(7,105) = 5.37, P = 0.002, suggesting that the effects of outcome probability on FRN amplitude differ in win and loss domain.

For win outcomes, tests of within-subjects contrasts revealed a significant linear main effect, F(1,15) = 32.90, P<0.001, and a significant quadratic, F(1,15) = 7.56, P = 0.015, suggesting that win-evoked FRN encode both reward probability and uncertainty, when examined separately. Consistent with the ANOVA analysis, regression analysis revealed that the win-evoked FRN (Fig. 2B) was significantly modulated by positive prediction error, *t(7)* = −8.20, *p*<0.001, and uncertainty prediction error, *t(7)* = 7.89, *p* = 0.001, with a coefficient of −2.596±0.32 and 2.234±0.28 for positive prediction error and uncertainty prediction error, respectively (Fig. 2E, in blue. Note, the outcome probability in this figure refers to the actual outcome frequency, as explained in the figure caption). The regression coefficient associated with positive prediction error indicated that the FRN had larger amplitudes for infrequent win feedback, whereas the regression coefficient associated with uncertainty prediction error indicated FRN amplitudes were larger for the win outcome with lower reward uncertainty. The proportion of the variance explained by the model was very high, with *R* ^2^ = 0.947, *p* = 0.001.

In the loss condition, tests of within-subjects contrasts revealed a significant linear main effect, F(1,15) = 9.71, P = 0.007, and a non-significant quadratic, F(1,15) = 2.94, P = 0.107, suggesting that loss associated FRN encode reward probability but not uncertainty. In consistent with the ANOVA analysis, regression analysis revealed that the loss-evoked FRN (Fig. 2C) was significantly modulated by negative prediction error, *t(7)* = 7.70, *p* = 0.001, with a coefficient of −4.795±0.62, but not by uncertainty prediction error (the coefficient was 1.011±0.56, *t(7)* = 1.81, p = 0.130). The proportion of the variance explained by the model was high, with *R* ^2^ = 0.93, *p* = 0.001 (Fig. 2E, in red). Note, the regression coefficients associated with reward prediction error were negative for both win-evoked FRN and loss-evoked FRN, suggesting that infrequent outcome evoked stronger negative-going FRN in both win and loss domains.

### Source analysis of the FRN

**Figure 3:**
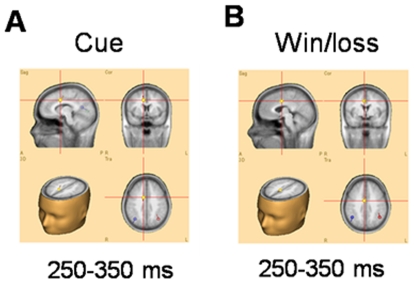
Sagittal, transversal, and coronal views of dipoles. Dipoles were superimposed on MRI-based head models for grand-average ERP waveforms in (A) cue phase and (B) outcome (win/loss) phase.

In the cue condition, the resulting five-source model accounts for the data with a residual variance of 4.86% (Fig. 3A) and the source of the cue-evoked FRN was located in the site of ACC (x = 10, y = 5, z = 37). In the win outcome condition, the resulting five-source model accounts for the data in the period 0 to 350 ms post onset of win feedback with a residual variance of 4.85% and the source of the win-evoked FRN was also located in the site of ACC (x = 5, y = −2, z = 37). The same model for the win condition also accounts for the ERP data in the loss condition with a residual variance of 4.74%, suggesting that win and loss ERPs have the same sources (Fig. 3B). Thus the dipole source analysis further indicated an involvement of the ACC in the rapid processing of reward probability and uncertainty signals.

## Discussion

In this study, the FRN, as an indicator of the ACC activity, was measured in a simple gambling task in which reward probability and uncertainty could be dissociated. We provided, for the first time to our knowledge, a quantitative analysis of the encoding of reward probability and uncertainty in the human ACC. Our results suggest that the cue-evoked FRN may encode reward probability and uncertainty. While both win and loss-related FRN amplitudes decreased as a function of outcome probability, only the win-related FRN but not the loss-related FRN was modulated by reward uncertainty. These results provide new insights into the functions of the ACC in reward decision making.

Previous ERP studies have examined the encoding of reward probability in the ACC. They only used limited number of probability values (i.e. 25%, 50%, and 75%) and yielded inconsistent findings [22], [24]–[26]. Two studies found that negative prediction errors evoked larger FRN amplitudes than positive prediction errors [22], [24]. While one study found that reward probability only modulated the win-evoked FRN, but not the loss-evoked FRN [25], another study found that reward probability modulated neither the win-evoked FRN nor the loss-evoked FRN [26]. The present study has two unique features that may allow us to overcome the limitations of previous studies and provide a more comprehensive analysis of the encoding of reward parameters in the ACC. First, the reward probability information was explicitly provided with a cue card and the feedback cannot be used to optimize decisions. Thus our design minimized the possible influence of asymmetric sensitivity to positive and negative outcomes in learning [27], [28]. Second, reward probability was varied over a wide range of probabilities with a sufficient number of intermediate values (every 12.5% from 0 to 100%) to ensure that reward probability and uncertainty were disassociated.

The first main finding of this study was that the amplitudes of the win- and loss-evoked FRNs all increased with outcome probability, indicating that positive and negative prediction errors were similarly encoded in the ACC. This finding challenges the hypothesis that the ACC activity mirrors the activity of the midbrain dopamine neurons in the encoding of reward prediction error in win and loss conditions [29], [30]. The reinforcement learning theory of the FRN proposes that the FRN reflects the impact of the midbrain dopamine signals on the ACC [29], [30]; the phasic changes in the midbrain dopamine activity are associated with fluctuations in the FRN amplitude and negative and positive prediction errors increase and decrease the FRN amplitude, respectively [29], [30]. The phasic decreases in dopamine inputs elicited by negative prediction errors give rise to the increased ACC activity that is reflected as larger FRN amplitudes. The phasic increases in dopamine signals elicited by positive prediction errors give rise to decreased ACC activity that is reflected as smaller FRN amplitudes. While a linear correlation between the negative prediction error and the FRN amplitude in this study is consistent with earlier ERP studies [22], [24], [31]–[33], the linear association of the larger FRN amplitude with larger (rather than smaller) positive prediction error is a novel finding, which suggests that positive prediction errors evoke a linear increase rather than decrease in the ACC activity. It has been found that negative feedback elicited a large FRN only when participants estimated they had responded correctly but not when they estimated they had responded erroneously [34]. Further, false-positive feedback presented after participants made large errors after erroneous trials elicited a significantly larger FRN than negative feedback [34]. Our study extends these previous findings by further showing a linear relationship between probability and FRN amplitude. Violation of reward magnitude expectation was also found to elicit larger FRN [35]. These results are consistent with recent single unit recording studies in monkeys and humans that found two groups of ACC neurons sensitive either to unexpected wins or losses [36]–[38]. Taken together, these findings support the notion that the ACC generally monitors violations in expectancy rather than negative feedback per se [34].

The second main finding was that reward uncertainty was encoded in the cue-evoked FRN and the win-evoked FRN. Uncertainty is crucial to decision making and attention based learning [1]. Different from monkeys' midbrain dopamine neurons that encode reward uncertainty by sustained and delayed signals [4], the present study showed that reward uncertainty signals were rapidly processed in the human ACC. This rapid encoding of uncertainty may reflect the need for a rapid motivational evaluation of the informativeness of stimuli. We found that larger cue-evoked FRN amplitudes were elicited by cues indicating high uncertainty in making reward prediction. This finding is consistent with the notion that the FRN reflects motivational evaluation of outcome since high uncertainty cues are less informative and thus less rewarding to participants. This finding is also consistent with an earlier fMRI study that also found stronger ACC activity to the uncertainty of reward cues during reward anticipation [15]. The uncertainty is resolved when outcomes are presented. The resolution of high uncertainty should be more informative and more rewarding than the resolution of low uncertainty. Indeed, for the win outcome, compared with wins following more certain cues, wins following uncertain cues are evaluated more positively, indicated by the decreased FRN amplitudes (i.e., more positive deflection). Our informativeness account is supported by the evidence that human ACC activity in the outcome monitoring phase is modulated by the volatility or uncertainty of the reward environment [13]. Taken together, these findings highlight the contribution of ACC in encoding uncertainty.

Some limitations in the present study are worth mentioning. First, in the outcome phase, the numbers of trials change with experimental conditions, raising the possibility that trial numbers may contribute to the FRN patterns. However, a recent study found that the FRN component rapidly stabilizes at 20 trials (or even 10 trials in one experiment) in healthy populations [39], indicating that increasing the number of trials after that would not significantly change the FRN amplitude. Second, although the objective reward probability associated with each cue card is explicit, different participants might perceive them differently. Moreover, participants may have irrational believe that their actions could influence outcomes [40], [41] and they may be overoptimistic about the chances of winning [34]. How the subjective probabilities might differ from objective probabilities is an interesting question for future studies. Third, our interpretation of associations between uncertainty and FRN amplitudes is speculative. The exact mechanisms reflected in the FRN amplitude/ACC activity are largely unknown. It is also currently unknown why the uncertainty effect was significant for the FRN in the win condition but not in the loss condition. Also, the informativeness account cannot explain other FRN findings, such as why the FRN is more negative for losses than for wins. Further computational model-based studies are needed to resolve this issue. Fourth, although the FRN has primarily been localized to the ACC [8], [42], [43], there is no direct evidence to link the ACC with the FRN. In fact, some studies have localized the FRN to the striatum [44], [45]. The mesocorticolimbic dopamine system, which includes the midbrain, striatum, orbital frontal cortex, and medial prefrontal cortex (e.g. ACC), has long been implicated in reward processing [46]. It is possible that the FRN also reflects reward processing in reward regions beyond the ACC [45]. Fifth, recent studies suggest that modulation of the FRN amplitudes results from the superposition on correct trials of a positive-going deflection, known as reward positivity [21], [47]–[49]. The reduction in the FRN amplitude could have resulted from superposition of the reward positivity that cancels out the FRN. Given that the present study was not designed to test these possibilities, further studies are necessary to examine the FRN using advanced methods such as principal components analysis (PCA) [49]. Our FRN findings at the cue phase could be driven by some peculiar experimental conditions, and they are in need of replication before conclusive arguments are made.

In summary, we demonstrate that reward probability and reward uncertainty can be processed rapidly and discretely in the human ACC at about 300 ms after stimulus presentation. An integrated processing of uncertainty and probability enables optimal inference and learning in a noisy and changeable environment. Current models of the FRN should thus be modified to take into account the uncertainty signal in the ACC.
